# Optimal Resource Management and Binary Power Control in Network-Assisted D2D Communications for Higher Frequency Reuse Factor

**DOI:** 10.3390/s19020251

**Published:** 2019-01-10

**Authors:** Devarani Devi Ningombam, Seokjoo Shin

**Affiliations:** Department of Computer Engineering, Chosun University, 309 Pilmun-daero, Dong-gu, Gwangju 61452, Korea; devaraninin@gmail.com

**Keywords:** device-to-device communications, uplink resource management, fractional frequency reuse, interference, quality of service, greedy heuristic search algorithm, binary power control scheme

## Abstract

Device-to-device (D2D) communications can be adopted as a promising solution to attain high quality of service (QoS) for a network. However, D2D communications generates harmful interference when available resources are shared with traditional cellular users (CUs). In this paper, network architecture for the uplink resource management issue for D2D communications underlaying uplink cellular networks is proposed. We develop a fractional frequency reuse (FFR) technique to mitigate interference induced by D2D pairs (DPs) to CUs and mutual interference among DPs in a cell. Then, we formulate a sum throughput optimization problem to achieve the QoS requirements of the system. However, the computational complexity of the optimization problem is very high due to the exhaustive search for a global optimal solution. In order to reduce the complexity, we propose a greedy heuristic search algorithm for D2D communications so as to find a sub-optimal solution. Moreover, a binary power control scheme is proposed to enhance the system throughput by reducing overall interference. The performance of our proposed scheme is analyzed through extensive numerical analysis using Monte Carlo simulation. The results demonstrate that our proposed scheme provides significant improvement in system throughput with the lowest computational complexity.

## 1. Introduction

Future cellular networks aim to deliver high data rates to meet the requirements for various services, with new communications paradigms such as device-to-device (D2D) communications. D2D communications is a standardized technology for Long Term Evolution-Advanced (LTE-A), which was introduced in the Third Generation Partnership Project (3GPP) Release 12 [1]. In D2D communications, nearby devices can communicate directly by reusing uplink cellular links. Thus, D2D communications can offload traffic from the evolved Node B (eNB), avoid collision, reduce transmission delay, and greatly improve the system performance [2]. This approach promotes the concept of the Internet of Things (IoT). The IoT comprises of large number of devices that have information sharing capability over wireless networks. In the D2D approach, communications can be generated in two ways: network-assisted communications and non-network-assisted communications [3]. Network-assisted D2D communications can be established with the aid of a radio access network. In LTE-A, the eNB provides information regarding the D2D pairs (DPs). Thus, the system obtains proximity devices information, which periodically updates the location of the devices. In the non-network-assisted D2D approach, communications can be independently established without a radio access network. As the main benefit of network-assisted D2D communications, the transmit power of users is periodically maintained. However, network-assisted D2D communications introduces new challenges into the traditional cellular networks when available resources are shared. The primary challenge lies in co-channel interference caused by DPs to traditional cellular users (CUs). Moreover, mutual interference between DPs also arises. Thus, to address the uplink interference issue, an efficient resource management algorithm for D2D communications is necessary. The allocation of uplink resources in D2D communications occurs either in overlay or underlay inband cellular networks [4,5]. In an overlay scenario, different resources are assigned to both DPs and CUs. This mechanism has negligible interference between CUs and DPs. However, spectral efficiency is not achieved in the network. On the other hand, in an underlay scenario, DPs and CUs share the same resources. Thus, spectral efficiency is achieved in the network. However, the main challenge faced by the network is the high uplink interference between CUs and DPs.

In order to alleviate the co-channel interference from DPs to traditional CUs, majority of the literature has focused on resource sharing methods. In [6], an efficient resource management scheme was proposed to maximize the spectrum efficiency by mitigating interference. In this study, the authors considered the transmission length of D2D communications based on transmission time slots. An optimization problem was formulated in [7], in which a two-stage resource management scheme was analyzed in terms of subcarrier assignment. The authors also analyzed a power control algorithm using the Lagrangian dual technique. In [8], the authors proposed a power management scheme for an underlay D2D communications in cellular networks. The problem was analyzed in two steps. First, proximity discovery was performed for D2D pairing, and second, direct communication between proximate devices was generated. In this study, the traditional CUs were defined as broadcasters and/or observers: a broadcaster advertises their connection-oriented information to establish D2D communications and an observer monitors the information for the connection. A mode selection method for D2D communications was presented in [9], aiming to improve the sum throughput and alleviate the average traffic delay. In [10], a resource management method was discussed to maintain the QoS of users by maintaining the transmission power of each DP in a cell. The capacity of the DPs was analyzed by defining the minimum signal-to-interference-noise ratio (SINR) demand of the DPs. The authors in [11] have proposed an optimal channel assignment technique for D2D communications underlaying cellular networks. In this study, a channel assignment algorithm was first proposed based on a dynamic programming method. This algorithm reduced the computational complexity of the study. Furthermore, the authors studied a cluster-based channel assignment problem to achieve a high successful transmission probability. Unfortunately, in the schemes in [6,7,8,9,10,11], the network models cannot precisely mitigate interference, and the available frequency is not fully utilized; thus, the systems are not spectrally efficient.

To attain a massive number of connected devices with minimal interference in D2D communications, we propose a resource management and power control scheme for an underlaying D2D communications using a fractional frequency reuse (FFR) scheme. Aiming for high spectrum efficiency, in our study, a network-assisted D2D communications scenario is considered. In this paper, we not only consider an FFR scheme but also consider a scalable frequency reuse method to achieve the best system performance. The primary contributions of this paper are as follows:

- First, we define a system architecture that allows multiple DPs to simultaneously reuse the same uplink cellular resource, enabling maximum spectrum utilization.
- We propose an optimal resource management and power control scheme for underlay D2D communications in cellular networks based on the FFR scheme with a scalable frequency reuse factor to mitigate interference induced by DPs to CUs. Statistical channel information for both CUs and DPs is assumed to evaluate the SINR. Then, we formulate the sum throughput maximization problem by introducing the SINR and power bound constraints.
- The major limitation in sum throughput optimization is the high computational complexity that results from an exhaustive search. Thus, the throughput maximization problem can be solved by using two methods, namely, a greedy heuristic method and a binary power control method. The greedy heuristic search algorithm can provide a suboptimal solution by performing a local search. This algorithm minimizes interference by assigning a matching subcarrier to DPs. Then, a binary power control scheme is introduced to maximize the system throughput by achieving a near-optimal solution. The main benefit of the binary power control scheme is that it does not require continuous channel state information (CSI) updates, thus reducing the computational complexity.
- We perform rigorous numerical analysis and simulations, and the results demonstrate that the proposed resource management and binary power control scheme outperforms the traditional D2D communications underlaying uplink cellular networks.

To our knowledge, this is the first approach to introduce both a greedy heuristic search algorithm and a binary power control scheme using the FFR technique to handle the sum throughput optimization problem for D2D communications in cellular networks.

The remainder of this paper is presented as follows: Section 2 explains the related works. In Section 3, we present the system model and problem formulation. Section 4 presents the proposed resource management and power control scheme. The resource management method is analyzed using a greedy heuristic search algorithm and a binary power control scheme. The results of performance analysis are provided in Section 5, and this paper is concluded in Section 6.

## 2. Related Works

The aforementioned works typically focused on resource management methods without employing a spectrum partition technique. The spectrum partition technique is also known as the FFR method, which partitioned the cell coverage into non-overlapping regions using directional antennas [12]. The FFR method can efficiently allocate the available resources among devices based on the channel coefficients of proximate devices. Thus, the FFR method mitigates interference between traditional CUs and DPs, improving the spectrum utilization in the network. A resource allocation scheme based on the FFR scheme was presented in [13]. In their work, the authors assumed a dynamic power control scheme and formulated a throughput maximization problem based on the heuristic search algorithm. In [14], a resource management method was discussed for an underlay D2D communications in cellular networks using an FFR scheme. In this paper, the authors proposed a non-orthogonal resource sharing scheme for an underlay D2D communications. A cell sectorization technique using three 120° directional antennas was considered to mitigate co-channel interference between users. Finally, a throughput maximization problem was developed based on SINR requirement and the upper and lower bound power levels. However, in their network, the frequency reuse factor is equal to one. In [15], an interference mitigation method for D2D wireless multimedia sensor networks was presented. In their study, an orthogonal frequency assignment technique was applied by dividing the entire cell area into six non-overlapping zones. In addition, a FFR technique was considered to improve the spectrum utilization. But, the optimal power management scenario was not achieved. A distance-constrained resource management method for an underlay D2D communications in cellular networks was proposed in [16]. D2D communications in a cell was categorized depend on the location of users. Moreover, the outage probability was analyzed based on the SINR. However, the methods in [15,16] cannot support simultaneous D2D connections for simultaneous communications. Therefore, these schemes are not effective for high-density networks.

A few studies have considered a frequency reuse factor much higher than one in D2D communications underlaying cellular networks. To accommodate the maximum number of DPs that can simultaneously communicate, a scalable frequency reuse factor for an underlay D2D communications was presented in [17]. In their study, blind admission control, a distributed admission control method and optimal admission control methods were analyzed to achieve the required QoS for both CUs and DPs in a cell. Additionally, an optimization problem was formulated based on the CSI to maximize the network frequency reuse, which results in higher system capacity. However, in their study, the network model could not obtain the optimal interference mitigation scenario due to the absence of an FFR scheme. A spectrum reuse method along with power control scheme was discussed in [18]. In this study, the Stackelberg game approach was analyzed for multi-sharing D2D communications to maximize the independent set of users that simultaneously reused the same subchannels. Therefore, the performance of the proposed scheme increases. In [19], the authors proposed a fast spectrum reuse and power control scheme for D2D communications. A Stackelberg power algorithm was derived, where DPs that are willing to connect are divided into groups. In their study, the analysis was performed in two steps: First, resource assignment was performed based on the maximum independent set to reduce the number of iterations, and second, resource assignment was performed using the Stackelberg power algorithm to reduce the computational complexity of the proposed method. A dynamic FFR scheme to mitigate intercell interference was presented in [20]. In this study, the authors proposed a non-orthogonal multiple access multicellular communication system to simultaneously share subchannels, which results in improved spectral efficiency. The disadvantage of their study is that the optimal interference mitigation scenario could not be achieved due to intercell interference.

## 3. System Model and Problem Formulation

### 3.1. System Model

**Figure 1:**
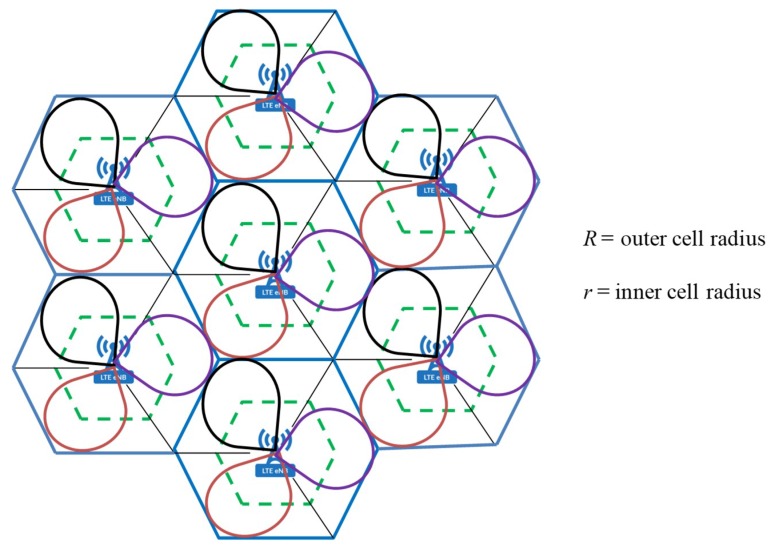
The multicell cellular network with cell sectorization using three-120° directional antennas.

In this paper, we focus on an uplink cellular network in which uplink resources are assigned to CUs by the eNB in an orthogonal manner, which avoids mutual interference among CUs. The main benefit of the uplink resource reuse phenomenon is that the eNB can exclusively coordinate the interference in a fully loaded cellular network [21]. We consider a regular multicell cellular network, where eNBs are placed at the center of the cells, as shown in Figure 1. It is also assumed that all cells are non-overlapping. The cells are divided into center zone with radius *r* and edge zone with radius *R,* and different subbands are assigned to each zone.

**Figure 2:**
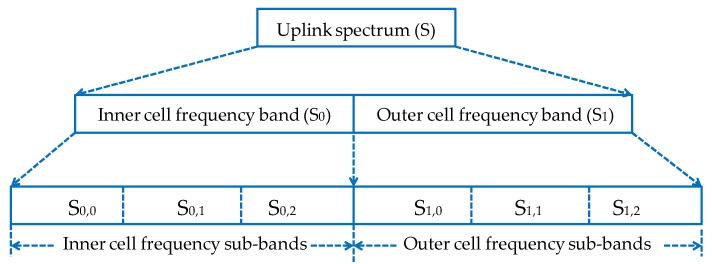
Uplink frequency partitioning using the FFR scheme.

As shown in Figure 2, the available uplink spectrum *S* is partitioned into two bands, namely, *S*_0_ and *S*_1_. *S*_0_ is the frequency band assigned to users located in the center zone, and *S*_1_ is the frequency band assigned to users located in the edge zone. Moreover, *S*_0_ and *S*_1_ are divided into six sub-bands, namely *S*_0,0_, *S*_0,1_, *S*_0,2_ and *S*_1,0_, *S*_1,1_, *S*_1,2_, respectively. For analysis, in our network architecture, we estimate that each cell has *M* DPs forming a set *D*, *D*={1, 2, 3, …, *M*}, coexisting with *N* CUs forming a set *C*, *C*={1, 2, 3, …, *N*}. Thus, there are *K* subcarriers forming a set *X*, *X=* {1, 2, 3, …, *K*}. A DP consists of a D2D transmitter and its corresponding receiver. All DPs and CUs follow a uniform distribution mechanism. Aiming at high spectrum utilization, it is assumed that DPs and CUs simultaneously share the same subchannels, and multiple DPs are allowed to reuse the same subchannel at a time. In order to mitigate uplink interference, we assume that DPs in the center cell region can reuse the resources of CUs located in the edge zone and vice versa. However, DPs in the center cell region cannot reuse the resources of CUs located in the center zone, and DPs located in the edge zone cannot reuse the resources of CUs located in the center zone.

In order to indicate the resource reuse factor, we define a binary variable $\delta_{m,n}^{k}$, where m ϵ D, n ϵ N and k ϵ X. When $\delta_{m,n}^{k}=1$, DP *m* can simultaneously reuse subcarrier *k* with CU *n*, otherwise $\delta_{m,n}^{k}=0$. In our proposed scheme, we have formulated the resource reuse factor (*F*) as follows:

(1) $$F=\sum_{n=1}^{N}\sum_{m=1}^{M}\delta_{m,n}^{k}+1.$$

Hence, the objective function is proportionate to maximizing $\sum_{n=1}^{N}\sum_{m=1}^{M}\delta_{m,n}^{k}$. This facilitates maximum resource reuse factor.

To simplify the analysis, we have made the following assumption. We consider that the cell structure is analogous to circle. Therefore, to analyze the resource reuse scenario, the probability density function (PDF) of users in center zone in terms of polar coordinates ($\rho_{in},\;\varphi$) is [22]:

(2) $$f\left(\rho_{in}\right)=\;\frac{2\left(\rho_{out}-\rho_{out}^{min}\right)}{{\left(r-\rho_{out}^{min}\right)}^{2}}$$

and

(3) $$f\left(\varphi \right)=\frac{3}{2\pi }\;,\;\forall \;0\leq \varphi \leq \frac{2\pi }{3}.$$

Therefore, the corresponding cumulative distribution function (CDF) is:

(4) $$\begin{array}{cc} P\left(\rho_{in}\right) & =\int_{\rho_{in}^{min}}^{r}f\left(\rho_{in}\right)d\rho_{in}\\ & =\int_{d_{mi\rho_{in}^{min}n}}^{r}\frac{2\left(\rho_{in}-\rho_{in}^{min}\right)}{{\left(r-\rho_{in}^{min}\right)}^{2}}d\rho_{in}\\ & =\frac{1}{{\left(r\;-\;\rho_{in}^{min}\right)}^{2}}{\left[2{\left(\rho_{in}\right)}^{2}-2\rho_{in}.\rho_{in}^{min}\right]}_{\rho_{in}^{min}}^{r}\\ & =\frac{2r}{\left(r\;-\;\rho_{in}^{min}\right)} \end{array}\}\rho_{in}^{min}\leq \;\rho_{in}<\;r.$$

Similarly, the PDF of users in edge zone in terms of polar coordinates ($\rho_{out},\;\varphi$)is:

(5) $$f\left(\rho_{out}\right)=\;\frac{2\left(\rho_{out}-\rho_{out}^{min}\right)}{{\left(R-\rho_{out}^{min}\right)}^{2}}$$

and

(6) $$f\left(\varphi \right)=\frac{3}{2\pi }\;,\;\forall \;0\leq \varphi \leq \frac{2\pi }{3}.$$

Therefore, the corresponding cumulative distribution function (CDF) is:

(7) $$\begin{array}{cc} P\left(\rho_{out}\right) & =\int_{\rho_{out}^{min}}^{R}f\left(\rho_{out}\right)d\rho_{out}\\ & =\int_{\rho_{out}^{min}}^{R}\frac{2\left(\rho_{out}-\rho_{out}^{min}\right)}{{\left(R-\rho_{out}^{min}\right)}^{2}}d\rho_{out}\\ & =\frac{1}{{\left(R\;-\;\rho_{out}^{min}\right)}^{2}}{\left[2{\left(\rho_{out}\right)}^{2}-2\rho_{out}.\rho_{out}^{min}\right]}_{\rho_{out}^{min}}^{R}\\ & =\frac{2R}{\left(R\;-\;\rho_{out}^{min}\right)} \end{array}\},\;\rho_{out}^{min}\leq \;\rho_{out}<\;R.$$

where $\rho_{in}^{min}$ and $\rho_{out}^{min}$ are the minimum allowable distance from the eNB to center zone and edge zone users.

### 3.2. Problem Formulation

To share subchannel resources between the CUs and DPs, it is important to analyze the transmission power management method. To apply the power management method while generating a D2D connections, the general form of transmit powers for CU *n* and DP *m* can be derived as follows [23]:

(8) $$P_{n}=\;P_{n}^{max}\cdot \;\frac{P_{N}}{l_{n,B}^{-\alpha }}$$

and

(9) $$P_{m_{t}}=\;P_{m_{t}}^{max}\cdot \;\frac{P_{N}}{l_{m_{t},m_{r}}^{-\alpha }},$$

respectively, where $P_{n}$ and $P_{m_{t}}$ represent the transmit power for CU *n* and D2D transmitter $m_{t}$, respectively, and $P_{n}^{max}$ and $P_{m_{t}}^{max}$ represent the maximal transmit powers of CU *n* and D2D transmitter $m_{t}$, respectively. $P_{N}$ represents the normalized power density, α denotes the path loss exponent, $l_{n,B}$ denotes the distances from the eNB to CU *n*, and $l_{m_{t},m_{r}}$ denotes the distance between D2D transmitter $m_{t}$ and receiver $m_{r}$. Hence, the received powers for CU *n* and DP *m* are as follows [24]:

(10) $$\begin{array}{c} P_{R_{n}}=\;P_{n}\cdot \;l_{n,B}^{-\alpha }\cdot {\left|h_{n,B}\right|}^{2}\\ =P_{n}^{max}\cdot \frac{P_{N}}{l_{n,B}^{-\alpha }}\;\cdot l_{n,B}^{-\alpha }.{\left|h_{n,B}\right|}^{2}\\ =\;P_{n}^{max}\cdot P_{N}\cdot {\left|h_{n,B}\right|}^{2} \end{array}\}$$

and

(11) $$\begin{array}{c} P_{R_{m}}=\;P_{m_{t}}\cdot \;l_{m_{t},m_{r}}^{-\alpha }\cdot {\left|h_{m_{t},m_{r}}\right|}^{2}\\ =P_{m_{t}}^{max}\cdot \;\frac{P_{N}}{l_{m_{t},m_{r}}^{-\alpha }}\;\cdot l_{m_{t},m_{r}}^{-\alpha }\cdot {\left|h_{m_{t},m_{r}}\right|}^{2}\\ =\;P_{m_{t}}^{max}\cdot P_{N}\cdot {\left|h_{m_{t},m_{r}}\right|}^{2} \end{array}\},$$

respectively, where $h_{n,B}$ represents the channel coefficient between the eNB and the CU *n* and $h_{m_{t},m_{r}}$ represents the channel coefficient between the D2D transmitter $m_{t}$ and receiver $m_{r}$. Therefore, we can calculate the overall power consumption of the system by formulating a power efficiency problem. The power efficiency can be defined as the ratio of the overall system throughput to the total power consumption.

#### 3.2.1. Channel Model

To evaluate the connection mode of users, we assume the Winner II B5f path loss (PL) model for urban areas. Winner II B5f is the most promising channel model that accounts both line of sight (LOS) and non-line of sight (NLOS) communication environments. Therefore, the PL model is expressed as [25]:

(12) $$PL_{DP}=57+23.5log_{10}\left(l\right)+23log_{10}\left(\frac{f}{5}\right),$$

where l denotes the distance between the transmitter and receiver in meters (30 m < *l* < 1.5 km) and *f* is the carrier frequency in GHz (2 GHz < *f* < 6 GHz).

#### 3.2.2. Interference Management

**Figure 3:**
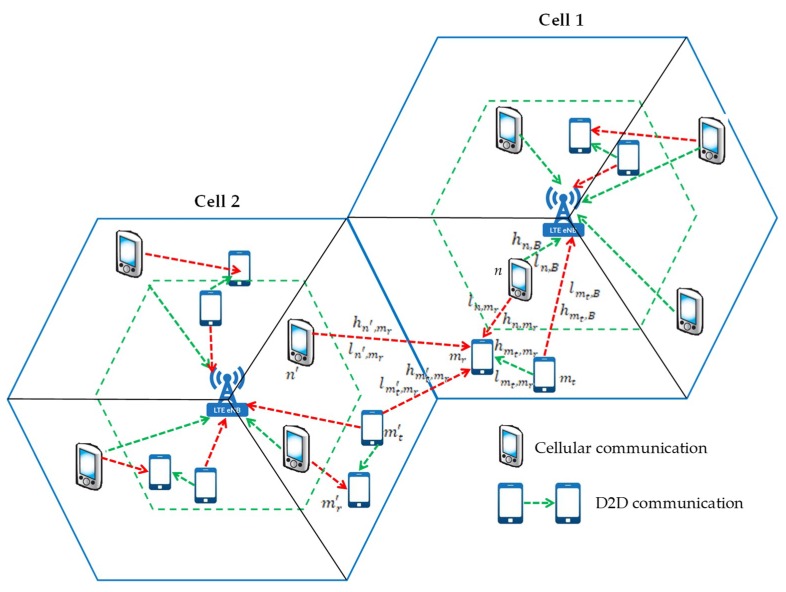
Demonstration of the interference between two neighboring cells, when a D2D communications is overlapped on a cellular resource.

In the network-assisted D2D communications, first the eNB collects CSI and required SINR information of all the users. Then, eNB calculates the transmit powers of CUs and DPs. In this subsection, we formulate the SINR problem which aims at minimizing the sum interference. The SINR is defined as the ratio of the power of a certain desired signal to the sum of interference power (from all other interfering devices) and noise power [26]. The uplink interference scenarios for a multicell cellular network are shown in Figure 3. Taking the cell 1 as target cell, in which the D2D transmitter $m_{t}$ communicates with D2D receiver $m_{r}$ located in edge zone by reusing subcarrier resource k of the CU *n* located in center zone. During data transmission between the D2D transmitter $m_{t}$ and receiver $m_{r}$, the possible uplink interference introduced in the network are, namely co-channel interference from D2D transmitter $m_{t}$ to eNB ($I_{B}^{m_{t}}$), co-channel interference from CU *n* to D2D receiver $m_{r}$ ($I_{m_{r}}^{n}$), interference from the D2D transmitter $m_{t}^{\prime }$ of neighboring cell 2 to eNB of cell 1 ($I_{B}^{m_{t}{}^{\prime }}$), interference from the CU $n^{\prime }$ of neighboring cell 2 to D2D receiver $m_{r}$ of cell 1 ($I_{m_{r}}^{n^{\prime }}$), and mutual interference between D2D receiver $m_{r}$ and neighboring cell D2D transmitter $m_{t}^{\prime }$ ($I_{m_{r}}^{m^{\prime }}$). Therefore, the SINRs of CU *n* and DP *m* on subcarrier *k* are:

(13) $$\gamma_{n}^{k}=\frac{P_{n}^{k}\cdot l_{n,B}^{-\alpha }\cdot |h_{n,B}{|}^{2}}{I_{B}^{m_{t}}+I_{B}^{m_{t}^{\prime }}+\sigma_{n}{}^{2}},\forall n\in C,\;m\in D,k\in X$$

and

(14) $$\gamma_{m}^{k}=\frac{\delta_{m,n}^{k}\cdot P_{m_{t}}^{k}\cdot l_{m_{t},m_{r}\cdot }^{-\alpha }|h_{m_{t},m_{r}}{|}^{2}}{I_{m_{r}}^{n}+I_{m_{r}}^{n^{\prime }}+I_{m_{r}}^{m_{t}^{\prime }}+\sigma_{n}{}^{2}},\forall n^{\prime }\in C,\;m_{t},m_{r},\;m_{t}^{\prime }\in D,k\in X,$$

respectively, where $P_{n}^{k}$ and $P_{m_{t}}^{k}$ represent the transmit power of CU *n* and D2D transmitter $m_{t}$ for channel *k,* respectively, and $\sigma_{n}$ is the noise power.

Thus, we define the interference terms as follows:

(15) $$I_{B}^{m_{t}}=\overset{M}{\underset{m=1}{\sum }}\overset{N}{\underset{n=1}{\sum }}\delta_{m,n}^{k}\cdot P_{m_{t}}^{k}\cdot l_{m_{t},B}^{-\alpha }\cdot |h_{m_{t},B}{|}^{2},\;$$

(16) $$I_{B}^{m_{t}^{\prime }}=\overset{M}{\underset{\begin{array}{c} m=1\\ m_{t}^{\prime }\neq m_{t} \end{array}}{\sum }}P_{m_{t}^{\prime }}^{k}\cdot l_{m_{t}^{\prime },B}^{-\alpha }\cdot |h_{m_{t}^{\prime },B}{|}^{2},$$

(17) $$I_{m_{r}}^{n}=\overset{M}{\underset{m=1}{\sum }}\overset{N}{\underset{n=1}{\sum }}P_{n}^{k}\cdot l_{n,m_{r}}^{-\alpha }\cdot |h_{n,m_{r}}{|}^{2},\;$$

(18) $$I_{m_{r}}^{n^{\prime }}=\sum_{m=1}^{M}\sum_{\begin{array}{c} n=1\\ n\neq n^{\prime } \end{array}}^{N}P_{n^{\prime }}^{k}\cdot l_{n^{\prime },m_{r}}^{-\alpha }\cdot |h_{n^{\prime },m_{r}}{|}^{2},$$

(19) $$I_{m_{r}}^{m_{t}^{\prime }}=\sum_{\begin{array}{c} m=1\\ m_{t}^{\prime }\neq m_{t} \end{array}}^{M}\sum_{n=1}^{N}\delta_{m,n}^{k}\cdot P_{m_{t}^{\prime }}^{k}\cdot l_{m_{t}^{\prime },m_{r}}^{-\alpha }\cdot |h_{m_{t}^{\prime },m_{r}}{|}^{2},$$

where $l_{m_{t},B}$ and $l_{m_{t}^{\prime },B}$ denote the distance from the eNB to D2D transmitter $m_{t}$ and D2D transmitter $m_{t}^{\prime }$, respectively, $l_{n,m_{r}}$, $l_{n^{\prime },m_{r}}$, and $l_{m_{t}^{\prime },m_{r}}$ denote the distance from D2D receiver $m_{r}$ to CU *n*, CU $n^{\prime }$, and D2D transmitter $m_{t}^{\prime }$, respectively. $h_{m_{t},B}$ and $h_{m_{t}^{\prime },B}$ denote the channel coefficient between the eNB and D2D transmitter $m_{t}$, and between the eNB and D2D transmitter $m_{t}^{\prime }$, respectively, $h_{n,m_{r}}$, $h_{n^{\prime },m_{r}}$, and $h_{m_{t}^{\prime },m_{r}}$ denote the channel coefficient between D2D receiver $m_{r}$ and CU *n*, between D2D receiver $m_{r}$ and CU $n^{\prime }$, and between D2D receiver $m_{r}$ and D2D transmitter $m_{t}^{\prime }$, respectively.

From Equations (13)–(19), the throughput of the system can be calculated using Shannon’s equation as follows:

(20) $$T=log_{2}\left(1+SINR\right).$$

Therefore, the sum throughput of the system can be expressed as:

(21) $$T=T_{n}+T_{m}.$$

From (20), we can derive the overall system throughput as follows:

(22) $$T=\sum_{m=1}^{M}\sum_{n=1}^{N}\left[log_{2}\left(1+\gamma_{n}^{k}\right)+log_{2}\left(1+\gamma_{m}^{k}\right)\right].$$

From Equations (13) and (14), Equation (22) becomes:

(23) $$T=\left\{\sum_{n=1}^{N}\sum_{m=1}^{M}\left[log_{2}\left(1+\frac{P_{n}^{k}\cdot l_{n,B}^{-\alpha }\cdot |h_{n,B}{|}^{2}}{I_{B}^{m_{t}}+I_{B}^{m_{t}^{\prime }}+\sigma_{n}{}^{2}}\right)+log_{2}\left(1+\frac{\delta_{m,n}^{k}\cdot P_{m_{t}}^{k}\cdot l_{m_{t},m_{r}}^{-\alpha }\cdot |h_{m_{t},m_{r}}{|}^{2}}{I_{m_{r}}^{n}+I_{m_{r}}^{n^{\prime }}+I_{m_{r}}^{m_{t}^{\prime }}+\sigma_{n}{}^{2}}\right)\right]\right\}.$$

However, due to the interference induced by DPs to CUs while sharing resources, the throughput of CUs is reduced. Therefore, we can calculate the spectral efficiency of CUs as follows [27]:

(24) $$E_{n}=\;log_{2}\left(1+\frac{P_{n}^{k}\cdot l_{n,B}^{-\alpha }\cdot |h_{n,B}{|}^{2}}{\sigma_{n}{}^{2}}\right)–log_{2}\left(1+\frac{P_{n}^{k}\cdot l_{n,B}^{-\alpha }\cdot |h_{n,B}{|}^{2}}{I_{B}^{m_{t}}+I_{B}^{m_{t}^{\prime }}+\sigma_{n}{}^{2}}\right)$$

Thus, the throughput gain of CUs is expressed as follows:

(25) $$G_{n}=\;log_{2}\left(1+\frac{P_{n}^{k}\cdot l_{n,B}^{-\alpha }\cdot |h_{n,B}{|}^{2}}{I_{B}^{m_{t}}+I_{B}^{m_{t}^{\prime }}+\sigma_{n}{}^{2}}\right)+log_{2}\left(1+\frac{\delta_{m,n}^{k}\cdot P_{m_{t}}^{k}\cdot l_{m_{t},m_{r}}^{-\alpha }\cdot |h_{m_{t},m_{r}}{|}^{2}}{I_{m_{r}}^{n}+I_{m_{r}}^{n^{\prime }}+I_{m_{r}}^{m_{t}^{\prime }}+\sigma_{n}{}^{2}}\right)-log_{2}\left(1+\frac{P_{n}^{k}\cdot l_{n,B}^{-\alpha }\cdot |h_{n,B}{|}^{2}}{\sigma_{n}{}^{2}}\right)$$

#### 3.2.3. Throughput Optimization

Since, we presumed that the eNB has global CSI of all the available CUs and DPs. Thus, the system can handle a peak power level. Therefore, the sum throughput optimization problem of the network can be formulated as follows:

(26) $$A1.\arg\underset{m\;\epsilon D,\;k\epsilon K}{\max}\overset{N}{\underset{n=1}{\sum }}F\left(log_{2}\left(1+\frac{P_{n}^{k}.l_{n,B}^{-\alpha }.|h_{n,B}{|}^{2}}{\sigma_{n}{}^{2}}\right)+\overset{C}{\underset{n=1}{\sum }}G_{n}\right)$$

subject to:

(27) $$\frac{P_{n}^{k}\cdot l_{n,B}^{-\alpha }\cdot |h_{n,B}{|}^{2}}{I_{B}^{m_{t}}+I_{B}^{m_{t}^{\prime }}+\sigma_{n}{}^{2}}\geq \gamma_{n_{min}}^{k},\;\forall n\in C,\;m_{t},m_{t}^{\prime }\in D,k\in X,$$

(28) $$\frac{\delta_{m,n}^{k}\cdot P_{m_{t}}^{k}\cdot l_{m_{t},m_{r}}^{-\alpha }\cdot |h_{m_{t},m_{r}}{|}^{2}}{I_{m_{r}}^{n}+I_{m_{r}}^{n^{\prime }}+I_{m_{r}}^{m_{t}^{\prime }}+\sigma_{n}{}^{2}}\geq \gamma_{m_{min}}^{k},\;\forall n\in C,\;m_{t},m_{t}^{\prime }\in D,k\in X,$$

(29) $$P_{n}^{min}\leq \;P_{n}^{k}\leq P_{n}^{max},\;\forall n\in C,k\in X,$$

(30) $$P_{m_{t}}^{min}\leq \;P_{m_{t}}^{k}\leq P_{m_{t}}^{max},\;\forall m_{t}\;\epsilon \;D,\;k\in X,$$

(31) $$\delta_{m,n}^{k}\;\epsilon \;\left\{0,1\right\},\;\forall \;m\;\epsilon \;D,\;n\;\epsilon \;C,\;k\in X,$$

where $\gamma_{n_{Th}}^{k}$ and $\gamma_{m_{Th}}^{k}$ denote the minimum SINR requirements of CU *n* and DP *m* using channel *k*, respectively. $P_{n}^{min}$ and $P_{m_{t}}^{min}$ are the minimal transmit power of CU *n* and D2D transmitter $m_{t}$, respectively. $P_{n}^{max}$ and $P_{m_{t}}^{max}$ represent the maximal transmit power of CU *n* and $P_{m_{t}}^{max}$, respectively. The constraints in Equations (27) and (28) imply that the SINRs of both CUs and DPs should be equal to or more than the minimum required SINR to achieve the QoS requirements. The constraints in Equations (29) and (30) guarantee that the transmit power of CUs and DPs should be within the upper and lower bound power levels. The constraint in Equation (31) guaranteed the versatility of resource reuse factor within the upper and lower bounds power level. This assures QoS requirements of the users by limiting the uplink interference.

However, during the CSI exchange between large number of users and eNB, a significant interference overhead is generated. From the optimization problem formulated in Equation (26), we can see that the problem statement aimed to find a global optimal solution with an exhaustive search. Therefore, we analyze the computational complexity of the throughput maximization problem. First, we calculate the throughput of traditional CUs before the integration of D2D communications. Therefore, the throughput maximization problem has computational complexity of O(N×K). Finally, we allocate the subcarrier resources to DPs, such that the resource reuse factor is greater than one. Hence, the complexity of the final step is O(M×K×F). Therefore, the overall computational complexity of the network is O(N×K)+O(M×K×F). We observed that the computational complexity of the network is very high, which is not practical. Moreover, the computational complexity of a network increases exponentially with problem size. Thus, a low-complexity approach suitable for practical D2D communications environment to reduce the interference overhead is proposed in Section 4.

## 4. Proposed Scheme

### 4.1. Greedy Heuristic Resource Management Scheme (GHRMS)

To determine the optimal solution, we discuss a local search-based resource management scheme known as the greedy heuristic resource management scheme (GHRMS). The GHRMS is a well-known method for solving optimization problems [28]. Therefore, the interference induced by CU *n* to D2D receiver $m_{r}$, interference induced by D2D transmitter $m_{t}$ to eNB, and mutual interference between DPs should be less than a certain threshold level. That is:

(32) $$G_{n,m_{r}}\leq G_{n,m_{r}}^{Th},\;\forall n\in C,m_{r}\;\epsilon \;D,$$

(33) $$G_{m_{t},B}\leq G_{m_{t},B}^{Th},\;\forall m_{t}\;\epsilon \;D,$$

and

(34) $$G_{m_{t}^{\prime },m_{r}}\leq G_{m_{t}^{\prime },m_{r}}^{Th},\;\forall m_{t}^{\prime },m_{r}\;\epsilon \;D,$$

where $G_{n,m_{r}}=l_{n,m_{r}}^{-\alpha }.|h_{n,m_{r}}{|}^{2}$ and $G_{m_{t},B}=l_{m_{t},B}^{-\alpha }.|h_{m_{t},B}{|}^{2}$ and $G_{m_{t}^{\prime },m_{r}}=l_{m_{t}^{\prime },m_{r}}^{-\alpha }.|h_{m_{t}^{\prime },m_{r}}{|}^{2}$. $G_{n,m_{r}}^{Th}$,$G_{m_{t},B}^{Th}$ and $G_{m_{t}^{\prime },m_{r}}$ are the predefined threshold channel gains. The pseudo code for the GHRMS is shown in Algorithm 1.

**Algorithm 1:** Greedy Heuristic Resource Management Scheme.**Initialization** Step 1: *C=* Set of CUs Step 2: *D*= Set of DPs Step 3: *X*= Set of uplink subcarriers Step 4: F = Resource reuse factor Step 5: $U_{k}\;=\left(U_{n},U_{m}\right)$ **Resource management** Step 6: Obtain all channel information for CUs and DPs Step 7: *n*=1 Step 8: **for** each *n*
$\epsilon U_{n}$
**do** Step 9:  **for** each *m*
$\epsilon U_{m}$
**do** Step 10:   **if**
$\delta_{m,n}^{k}=1$. **then** Step 11:   **if**
$G_{n,m_{r}}\leq G_{n,m_{r}}^{Th},\;G_{m_{t},B}\leq G_{m_{t},B}^{Th}$ and $G_{m_{t}^{\prime },m_{r}}\leq G_{m_{t}^{\prime },m_{r}}^{Th}$
**then** Step 12:    Calculate $\gamma_{n}^{k}$ and $\gamma_{m}^{k}$ Step 13:    Decline all matching subcarrier assignments and compute the QoS requirements with the current SINR value Step 14:    Solve A1 Step 15:   **else** Step 16:    Check $\delta_{m,n}^{k}$ Step 17:    Solve the subcarrier assignments problem to obtain the minimum interference solution Step 18:   **end** Step 19:   **end** Step 20:  **end** Step 21: **end**

In every iteration of the GHRMS, it finds the optimal matching of subcarriers between CUs and DPs to maximize the resource reuse factor *F*. In this algorithm, in Step 5, we first define $U_{k}=\left(U_{n},U_{m}\right)$ as the function of CUs $U_{n}$ and DPs $U_{m}$ that introduces the least interference among them. At the initiation of resource management process, the eNB gathers the channel information for all CUs and DPs in a cell (Step 6). From Step 7 to Step 9, starting from *n*=1, the algorithm iterates through all CUs. For all *n* and *m*, the GHRMS searches the DPs that can reuse cellular resources. In Step 10, if DPs can reuse CUs resources, then Step 11 presents the benchmarks for selecting the users that can reduce the uplink interference induced due to the integration of D2D communications into the traditional cellular networks. In Step 12, based on the channel conditions, we calculate the SINRs for CUS and DPs. Step 13 declines the remaining matching subcarrier resource assignment and computes the QoS requirements with the current SINRs. In Step 14, we solved the throughput maximization problem with higher resource reuse factor. Else the state of the resource reuse condition is rechecked in Step 16. Finally, in Step 17, we continue the matching for subcarrier resource assignment until the benchmark is achieved. This procedure is repeated until all subcarrier resources of the CUs have been assigned to DPs. In the GHRMS, the set of DPs included in the previous search for an optimal solution is removed from the remaining search procedures. This step reduces interference and overcomes the complexity of the search procedure.

### 4.2. Binary Power Control Scheme (BPCS)

Here, we present an analytical characterization of the power control scheme to achieve distributed solutions with low computational complexity. The use of the binary power control scheme (*BPCS*) for network-assisted multicell cellular networks is well known [29,30]. This approach can achieve a near-optimal solution when multiple DPs simultaneously reuse the same cellular resource in a multicell communication environment, thus accommodating a high system capacity. To solve the optimization problem formulated in Equation (26), we can reformulate the throughput problem for CU *n* and DP *m* as follows:

(35) $$T_{n}^{*}=\;\frac{1}{C}\overset{C\;}{\underset{n=1}{\sum }}log_{2}\left(1+\gamma_{n}^{k}\right).$$

and

(36) $$T_{m}^{*}=\;\frac{1}{F}\sum_{m=1}^{D\;}log_{2}\left(1+\gamma_{m}^{k}\right),$$

respectively, where $T_{n}^{*}$ and $T_{m}^{*}$ represent the optimal throughput for CUs and DPs, respectively.

Therefore, the transmit powers of CUs $\left(P_{1}^{1},\;\ldots ,\;P_{N}^{K}\right)$ and DPs $\left(P_{1}^{1},\;\ldots ,\;P_{M}^{K}\right)$ should satisfy the following conditions:

(37) $$A2.\left(P_{1}^{1},\;\ldots ,\;P_{N}^{K}\right)=\arg\underset{n\;\epsilon C,\;k\in X}{\max}\overset{C\;}{\underset{n=1}{\sum }}\left[T_{n}^{*}\right]$$

(38) $$A3.\left(P_{1}^{1},\;\ldots ,\;P_{M}^{K}\right)=\arg\underset{m\;\epsilon \;D,\;k\;\in \;X}{\max}\overset{D\;}{\underset{m=1}{\sum }}\left[T_{m}^{*}\right]$$

subject to:

(39) $$G_{n,B}\leq G_{n,B}^{Th},\;\forall n\in C,$$

(40) $$G_{m,B}\leq G_{m,B}^{Th},\;\forall m\epsilon D,$$

(41) $$P_{n}^{min}\leq P_{n}^{k}\leq P_{n}^{max},\;\forall n\in C,k\in X,$$

(42) $$P_{m_{t}}^{min}\leq P_{m_{t}}^{k}\leq P_{m_{t}}^{max},\;\forall m_{t}\epsilon D,\;k\in X,$$

(43) $$\delta_{m,n}^{k}\epsilon \left\{0,1\right\},\;\forall \;m\;\epsilon \;D,\;n\;\epsilon \;C,\;k\in X.$$

The pseudo code for the *BPCS* is shown in Algorithm 2. Aiming at a higher throughput with large reuse factor, the channel conditions are analyzed for CUs and DPs. In *BPCS*, the transmission is initiated only if the channel quality is sufficient, i.e., $G_{n,B}\leq G_{n,B}^{Th}$ and $G_{m,B}\leq G_{m,B}^{Th}$. This constraint implies that the smaller values of $G_{n,B}$ and $G_{m,B}$ minimize interference between CUs and DPs, resulting in higher throughput and spectral efficiency. Therefore, Algorithm 2 is applied to control the transmit power of CUs and DPs that have a higher channel gain for the eNB and low interference. The transmit power can be increased until the required SINR is no longer satisfied. When the maximum user power has been reached and the required SINR value is achieved, the process stops.

**Algorithm 2:** Binary Power Control Scheme.**Initialization** Step 1: *N* = Set of CUs Step 2: *M*= Set of DPs Step 3: *K*= Set of uplink subcarriers Step 4: $\delta_{m}^{k}$= Resource reuse factor Step 5: $U_{k}=\left(U_{n},U_{m}\right)$ Step 6: $G_{n,B}\leq G_{n,B}^{Th}$ Step 7: $G_{m,B}\leq G_{m,B}^{Th}$ Step 8: $\delta_{m,n}^{k}\;\epsilon \;\left\{0,1\right\}$ Step 9: $P_{n}^{k}=P_{n}^{max}$, $P_{m_{t}}^{k}=P_{m_{t}}^{max}$ **Power Control Algorithm** Step 10: *n*=1 Step 11: **for** each *n*
$\epsilon U_{n}$
**do** Step 12:   **for** each *m*
$\epsilon U_{m}$
**do** Step 13:   **if**
$T_{n}\leq T_{n}^{*}$ and $T_{m}\leq T_{m}^{*}$
**then** Step 14:   $P_{n}^{k}\leftarrow P_{n}^{max}$ and $P_{m_{t}}^{k}\leftarrow P_{m_{t}}^{max}$ Step 15:   **else**
$P_{n}^{k}\leftarrow P_{n}^{max}$ and $P_{m_{t}}^{k}\leftarrow P_{m_{t}}^{min}$ Step 16:   **end** Step17:  **end** Step 18: **end**

### 4.3. Complexity Analysis of the Proposed Scheme

The proposed resource management and binary power control scheme has computational complexity of O[K{N+(M×F)}] for estimating resource reuse partner for all CUs and DPs. The computational complexity of the proposed scheme is relatively small. Hence, the proposed scheme reduces the implementation cost by proper deigning D2D communications. Moreover, as the value of F increases, system capacity of our proposed scheme increases significantly. We can conclude that for real system deployment, the proposed resource management and power control scheme potentially increase the system capacity with least interference overhead.

## 5. Simulation Results and Discussion

In this section, we first present the simulation environment used for our analysis and then evaluate the simulation results.

### 5.1. Simulation Environment

Here, we provide the simulation environment to evaluate the performance of the proposed scheme. We consider an uplink network environment with seven cellular cells, each with the eNB placed at the center. The CUs and DPs are considered to be uniformly distributed in each cell with Rayleigh distributed small-scale fading. The network assumes log-normal shadowing effect with standard deviation of 8dB and also assumes a path-loss exponent of 4. The main simulation parameters are listed in Table 1, and other simulation parameters were selected based on the 3GPP LTE regulation [31]. We evaluate the performance using Monte Carlo simulation with a total of 10,000 iterations. We compare the performance of the proposed method in terms of the system capacity, spectrum efficiency, and transmit power.

### 5.2. Simulation Results and Discussion

This subsection presents a comparative performance analysis of the resource management method without FFR scheme (RRM), resource management with greedy heuristic scheme (RM-WGHRMS) and resource management with binary power control scheme (RM-WBPCS).

**Figure 4:**
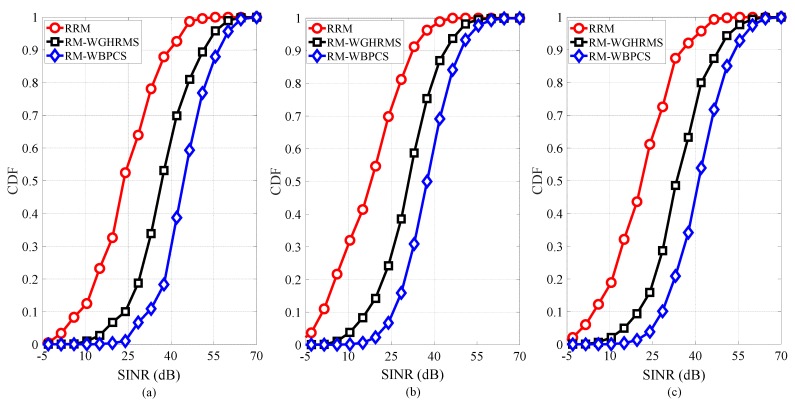
CDF of the SINR for (**a**) DPs, (**b**) CUs, and (**c**) the overall system.

Figure 4 shows the CDF of the SINR for DPs, CUs, and the overall system. We can see from Figure 4a that the GHRMS and BPCS allocate uplink resources to DPs that fulfill the SINR requirements. Thus, the SINR of DPs in our proposed method is higher than those in both resource management method without FFR scheme and resource management method with GHRMS. However, Figure 4b shows that the SINR of CUs in our proposed method is less than the SINR of DPs. This is because of the matter that CUs experiences interference from DPs. Finally, Figure 4c shows that the overall system SINR distribution of our proposed scheme achieves the highest value. We observed that the proposed scheme outperforms the other schemes in terms of SINR.

**Figure 5:**
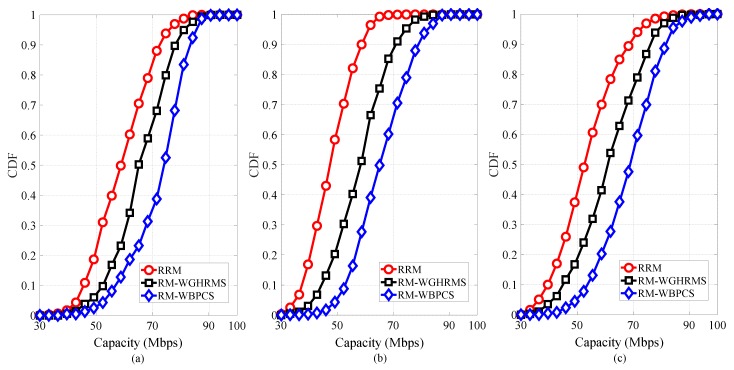
Total achievable capacity for (**a**) DPs, (**b**) CUs, and (**c**) the overall system.

Figure 5 presents the achievable capacity distribution for DPs, CUs, and the overall system. From Figure 5a, we can denote that the capacity distribution of DPs for our proposed scheme has the best performance. In contrast, from Figure 5b, we can observe that the capacity distribution of CUs is less than that of DPs. This result arises from the fact that the CUs experienced interference from co-channel DPs. In addition, we can observe from Figure 5c that the proposed scheme has the best capacity among existing schemes, since a larger frequency reuse factor increases the capacity of the system. Moreover, the results depicted in Figure 5 show that the FFR scheme assures the QoS requirements for DPs and CUs and achieves the optimal resource reuse partner between CUs and DPs.

**Figure 6:**
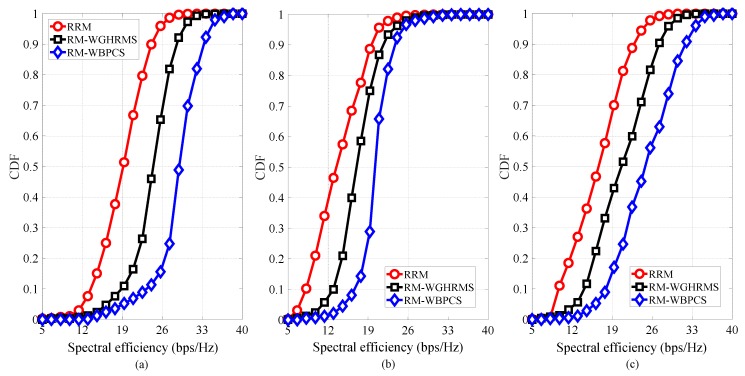
CDF of the spectral efficiency for (**a**) DPs, (**b**) CUs, and (**c**) the overall system.

The CDFs of the spectral efficiency for DPs, CUs, and the overall system are shown in Figure 6, respectively. Figure 6a shows that our proposed scheme has the best spectral efficiency for DPs among the existing schemes. Moreover, Figure 6b demonstrates that the proposed scheme yields a higher spectral efficiency for CUs compared to the other schemes. In addition, we can see from Figure 6c that our proposed scheme attains the highest overall system spectral efficiency; since the proposed resource management with GHRMS combined with the BPCS increases the spectrum utilization of the cell.

**Figure 7:**
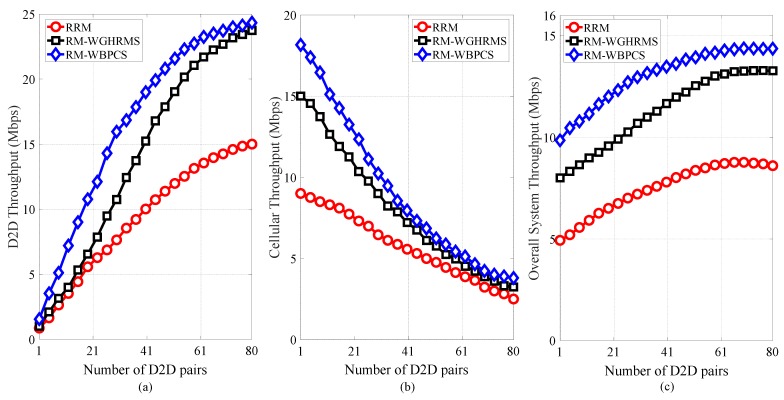
Average throughput analysis with varying numbers of available DPs for (**a**) DPs, (**b**) CUs, and (**c**) the overall system.

In Figure 7, the average throughput of our proposed scheme is compared with existing schemes. Figure 7a demonstrates that the throughput of DPs increases significantly with varying number of DPs. In contrast; Figure 7b shows that the throughput of CUs decreases dramatically with increasing DP number, which occurs because an increased DP number generates high uplink interference for traditional CUs. But, we can see from Figure 7c that the overall system throughput increases with varying number of DPs and that our proposed scheme outperforms the other schemes. The throughput increases with the increase of number of active DPs in a cell at first, then after reaching a peak number of affordable active DPs, the throughput saturates. We can see from Figure 7c that, after the number of active DPs exceeds 65, the throughput saturates.

**Figure 8:**
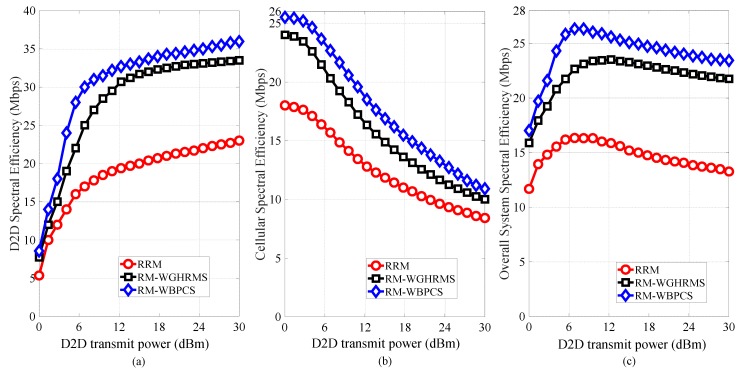
Spectral efficiency analysis with varying transmit power consumption of D2D for (**a**) DPs, (**b**) CUs, and (**c**) overall system.

Figure 8 shows the spectral efficiency for varying D2D transmit power. We can observe from Figure 8a that the spectral efficiency DPs increases with increasing D2D transmit power. In contrast, we can see from Figure 8b that the spectral efficiency of the CUs decreases with increasing D2D user transmit power, which occurs because a higher D2D transmit power increases the uplink interference for CUs. Moreover, Figure 8c shows that our proposed scheme yields the highest spectral efficiency among the studied schemes. We can see from Figure 8c that the spectral efficiency increases promptly as the D2D transmit power increases. However, after the D2D transmit power exceeds 11 dBm, the spectral efficiency decreases.

## 6. Conclusions

We have performed a brief analysis on the interference scenarios and techniques to mitigate the interference for underlay D2D communications in cellular networks. To mitigate the uplink interference and improve the overall system throughput, we have proposed an optimal resource management and power control scheme with cell sectorization method. We also considered the FFR technique, which allows fractional reuse of resources between the traditional CUs and DPs in a non-orthogonal fashion. In the proposed scheme, the resource reuse factor is analyzed by considering a binary random variable. Then, we formulated an optimization problem for multiple DPs simultaneously sharing the same cellular resources to fulfill the required QoS of both CUs and DPs. We analyzed the proposed scheme in two steps. We first solved a greedy heuristic algorithm with a local search scenario to overcome the complexity of the resource reuse pairing phenomenon. Then, we proposed a binary power control scheme to maximize the system capacity for large scale networks. We conducted extensive simulations with different parameters such as SINR, system capacity, spectral efficiency, and transmit power of DPs. The results indicated that our proposed technique achieves the highest capacity and spectral efficiency, and reduces the co-channel interference generated by the integration of D2D communications. As future work, our proposed method can be extended by considering the downlink resource reuse scenario for D2D communications.

## Figures and Tables

**Table 1:** Primary simulation parameters.

Parameter	Value
Cell outline	Hexagonal framework
Number of cells	7
Noise power density	−174 dBm/Hz
Distance between D2D transmitter and receiver	1~70 m
Carrier frequency	2 GHz [31]
Uplink bandwidth	5 MHz [31]
Path-loss exponent	4
Antenna type	120° directional antenna
Number of CUs per cell	65
Number of DPs per cell	80
Number of iterations	10,000

## References

[1] (2013). 3GPP TR 23.703 v0.5, Rel 12, in Technical Specification Group Services and System Aspects, Study on Architecture Enhancements to Support Proximity Services (ProSe). https://www.etsi.org.

[2] Wei L., Hu R.Q., Qian Y., Wu G. (2014). Enable device-to-device communications underlaying cellular networks: Challenges and research aspects. IEEE Commun. Mag..

[3] Andreev S., Pyattaev A., Johnsson K., Galinina O., Koucheryavy Y. (2014). Cellular traffic offloading onto network-assisted device-to-device connections. IEEE Commun. Mag..

[4] Katsinis G., Tsiropoulou E.E., Papavassiliou S. (2017). Multicell Interference Management in Device to Device Underlay Cellular Networks. Future Internet.

[5] Katsinis G., Tsiropoulou E.E., Papavassiliou S. (2017). Joint Resource Block and Power Allocation for Interference Management in Device to Device Underlay Cellular Networks: A Game Theoretic Approach. Mob. Netw. Appl..

[6] Phunchongharn P., Hossain E., Kim D.I. (2013). Resource allocation for device-to-device communications underlaying LTE-advanced networks. IEEE Wirel. Commun..

[7] Zhang Y., Li F., Al-qaness M.A.A., Luan X. (2017). A resource allocation scheme for multi-D2D communications underlaying cellular networks with multi-subcarrier reusing. Appl. Sci..

[8] Pratas N.k., Popovski P. Network-assisted device-to-device (D2D) direct proximity discovery with underlay communication. Proceedings of the Global Communications Conference (GLOBECOM).

[9] Zhao J., Chai K.K., Chen Y., Schormans J., Alonso-Zarate J. (2017). Joint mode selection and resource allocation for machine-type D2D links. Trans. Emerg. Telecommun. Technol..

[10] Yu B., Zhu Q. A QoS-based resource allocation algorithm for D2D communication underlaying cellular networks. Proceedings of the2016 Sixth International Conference on Information Science and Technology (ICIST).

[11] Novlan T.D., Ganti R.K., Ghosh A., Andrews J.G. (2011). Analytical Evaluation of Fractional Frequency Reuse for OFDMA Cellular Networks. IEEE Trans. Wirel. Commun..

[12] Wang R., Zhang J., Song S.H., Letaief K.B. (2016). Optimal QoS-Aware Channel Assignment in D2D Communications with Partial CSI. arXiv.

[13] Jiang F., Wang B.-C., Sun C.-Y., Liu Y., Wang X. (2016). Resource allocation and dynamic power conntrol for D2D communication underlaying uplink multi-cell networks. Wirel. Netw..

[14] Ningombam D.D., Shin S. (2018). Non-Orthogonal Resource Sharing Optimization for D2D Communication in LTE-A Cellular Networks: A Fractional Frequency Reuse-Based Approach. Electronics.

[15] Kim J., Kim T., Noh J., Cho S. (2018). fractional frequency reuse scheme for device-to-device communication underlaying cellular on wireless multimedia sensor networks. Sensors.

[16] Ningombam D.D., Shin S. (2018). Distance-constrained outage probability analysis for device-to-device communications underlaying cellular networks with frequency reuse factor of 2. Computers.

[17] Verenzuela D., Miao G. (2017). Scalable D2D Communications for Frequency Reuse gt; gt; 1 in 5G. IEEE Trans. Wirel. Commun..

[18] Chen K.-Y., Kao J.-C., Ciou S.-A., Lin S.-H. (2017). Joint Spectrum Reuse and Power Control for Multi-Sharing Device-to-Device Communication. arXiv.

[19] Lin S.-H., Chen K.-Y., Kao J.-C., Hsiao Y.-F. Fast Spectrum Reuse and Power Control for Device-to-Device Communication. Proceedings of the 2017 IEEE 85th Vehicular Technology Conference (VTC Spring).

[20] Mehmood K., Niaz M.T., Kim H.S. (2018). Dynamic fractional frequency reuse diversity design for intercell interference mitigation in nonorthogonal multiple access multicellular networks. Wirel. Commun. Mob. Comput..

[21] Doppler K., Rinne M.P., Janis P., Ribeiro C., Hugl K. Device-to-Device Communications; Functional Prospects for LTE-Advanced Networks. Proceedings of the2009 IEEE International Conference on Communications Workshops.

[22] Alouini M., Goldsmith A.J. (1999). Area spectral efficiency of cellular mobile radio systems. IEEE Trans. Veh. Technol..

[23] He A., Wang L., Chen Y., Wong K., Elkashlan M. (2017). Spectral and Energy Efficiency of Uplink D2D Underlaid Massive MIMO Cellular Networks. IEEE Trans. Commun..

[24] Banagar M., Maham B., Popovski P., Pantisano F. (2016). Power Distribution of Device-to-Device Communications in Underlaid Cellular Networks. IEEE Wirel. Commun. Lett..

[25] Hamid M., Kostanic I. (2013). Path Loss Models for LTE and LTE-A relay stations. Univ. J. Commun. Netw..

[26] Afroz F., Subramanian R., Heidary R., Sandrasegaran K., Ahmed S. (2015). SINR, RSRP, RSSI and RSRQ Measurements in Long Term Evolution Networks. Int. J. Wirel. Mob. Netw..

[27] Hwang Y., Park J., Sung K.W., Kim S.-L. (2015). On the throughput gain of device-to-device communications. ICT Express.

[28] Wang X., Lv S., Wang X., Zhang Z. (2018). Greedy heuristic resource allocation algorithm for device-to-device aided cellular systems with system level simulation. KSII Trans. InternetInf. Syst..

[29] Silva J.M.B., Fodor G. (2015). A binary power control scheme for D2D communications. IEEE Wirel. Commun. Lett..

[30] Memmi A., Rezki Z., Alouini M. (2017). Power control for D2D underlay cellular networks with channel uncertainty. IEEE Trans. Wirel. Commun..

[31] 3GPP Evolved Universal Terrestrial Radio Access (E-UTRA) and Evolved Universal Terrestrial Radio Access Network (E-UTRAN). https://www.etsi.org.

